# Health Communication Campaign Performance During the HEALing Communities Study: Cross-Sectional Examination of Digital Advertising Methods

**DOI:** 10.2196/75458

**Published:** 2026-04-01

**Authors:** Nicky Lewis, Jennifer Reynolds, Diane Krause, Philip M Reeves, Jamie Luster, Michael D Stein, Sharon L Walsh, Amy Farmer, Michelle R Lofwall, Monica F Roberts, Hilary L Surratt, Brooke N Crockett, Kara Stephens, Kelli Bursey, Kristin Mattson, Michael D Slater

**Affiliations:** 1 Indiana University Bloomington, 601 E Kirkwood Avenue, Bloomington, IN, 47405, United States, 1 812 855 6106; 2 Health and Resilience Strategies, Oak Ridge Associated Universities, Oak Ridge, TN, United States; 3 The Ohio State University College of Medicine, Columbus, OH, United States; 4 Boston University School of Public Health, Boston, MA, United States; 5 University of Kentucky, Lexington, KY, United States

**Keywords:** communications, opiate overdose, social stigma, naloxone, methadone, buprenorphine evidence-based practice

## Abstract

**Background:**

Research on the effectiveness of digital health campaign strategies is lacking. Understanding performance outcomes is essential for the successful implementation of campaigns. Two studies examined platforms, tactics, and content of digital health campaigns using paid media performance data.

**Objective:**

This analysis compared 2 digital advertising methods (social media and banner or display) using click-through rate (CTR) and cost-per-click (CPC) as performance measures. Performance differences by state, community type, message approach, format, and image type were assessed. CTR and CPC served as measures in determining performance differences between social media and banner or display.

**Methods:**

This cross-sectional secondary analysis examined campaign performance for the HEALing (Helping to End Addiction Long-Term) Communities Study, which served 85,875,105 impressions. Data were collected from media buy reports, entered into templates that included method (display or banner and social media) and key performance indicators (impressions, clicks, and media spend), and CTR and CPC were calculated. Study 1 assessed differences in CTR and CPC for social media and banner or display by state (KY, NY, MA, and OH) and community type (urban and rural). Study 2 assessed differences in CTR for social media and banner or display by state (KY, NY, MA, and OH), community type (urban and rural), message approach (testimonial and information-based), format (motion graphic or graphics interchange format, video, and static image), and image type (local and stock). Separate analyses were conducted for each advertising method.

**Results:**

Study 1 found significant differences between advertising methods, where social media had higher CTR compared to banner or display. Social media had a significant main effect for state, where OH had the highest CTR. There was a statistically significant difference in CPC based on advertising method, where social media had a lower CPC compared to banner or display. Social media had a significant main effect for state, where OH had the lowest CPC. Banner or display had a significant main effect for state and community type, where OH and urban communities had the highest CPC. Study 2 found significant differences between advertising methods, where social media had higher CTR than banner or display. For social media, urban communities, static format, and local spokespersons had the highest CTR. There were significant differences between all pairs of states, where OH had the highest CTR. For display or banner, static format and local spokespersons had the highest CTR.

**Conclusions:**

This analysis provides guidance for digital health campaigns. It examined the performance of opioid use disorder campaigns using CTR and CPC measures, demonstrating utility in future campaign evaluations. Social media was more related to stimulating responses to campaign messages compared to banner or display. State-to-state variations emphasized the importance of message pilot testing. Using local spokespersons versus stock spokespersons is recommended.

## Introduction

### Overview

Studies that examine the platforms (ie, banner or display and social media), tactics (ie, campaign strategy), and content (ie, campaign materials) that influence health communication campaign effectiveness are limited [1,2]. There are few published empirical reports on the comparative effectiveness of online advertising platforms in regard to online public health messaging content [3]. The current research effort examines the online advertising tactics, platforms, and messaging across the communication campaigns of the HEALing (Helping to End Addiction Long-Term) Communities Study (HCS) using paid media performance data.

### Background and Rationale

Examinations of the effectiveness of health communication campaigns are minimal and essentially absent in the domain of opioid use disorder (OUD); see Rath et al [4] for an exception. Most research on the effectiveness of health communication campaigns is centered on tobacco cessation efforts, including the Tips from Former Smokers campaign [5]. Durkin et al [6] highlight the potential of digital and social media in enhancing campaign effectiveness. Digital and social media may be particularly important when producing opioid-related health campaigns because of the stigmatized nature of opioid use. This is because digital and social media can often be consumed in private or semiprivate locations and on one’s individual media device (eg, tablet and smartphone), reducing the likelihood of others observing the content.

Most opioid-related campaigns focus on prevention or stigma reduction; some target the overdose reversal medication naloxone, but few focus on treatment with medications for opioid use disorder (MOUD) [7]. These campaigns frequently communicate to the general public rather than targeting messaging to priority audiences who could most benefit, such as individuals and loved ones affected by OUD and sectors that may influence local programs and policies regarding substance use, including criminal or legal services, emergency medical services, local government officials, faith-based leaders, and media [8]. Geographical, regional, and cultural differences can also contribute to varying outcomes associated with campaigns as well as the way campaigns are managed and the materials used [9]. Moreover, many opioid-related health campaigns fail to conduct formative research to inform audience segmentation and campaign messaging tactics and content or to use evidence-based practices for campaign development, implementation, and evaluation [7].

Although data regarding widespread opioid-related campaign effectiveness are limited, some evidence can be gleaned from smaller-scale message testing studies. Visual campaigns about stigmatizing language and MOUD treatment, combined with narrative vignettes from either people with lived experience experiencing OUD or of health care administrators, resulted in a significant reduction in stigmatizing attitudes among a large survey of health care providers [10]. Focus groups of young adults who use illicit drugs in Illinois provided evidence that effective campaigns should highlight the risk of fentanyl exposure, fill in gaps about fentanyl knowledge, form a personal connection in messaging, and use realistic details and images [11]. Among a sample of college students without personal OUD experience, a blog-style testimonial about an individual in recovery who used MOUD followed by abstinence-based treatment resulted in strong perceptions of the character’s fluidity and predicted stigma reduction [12]. These findings suggest the importance of personalized messaging with a testimonial-driven element, consistent with the literature on engagement with digital advertisements in general. Both personal relevance, defined as the process through which viewers determine if an advertisement or message relates to their personal goals or values, and social relevance, a situational element involving likes, comments, or other engagement with advertisements or messages by one’s social network, can lead to increased engagement [13].

### HCS Campaigns

The HCS was a multisite, parallel-group, community-level cluster randomized wait-listed trial supported by the National Institutes of Health (NIH) and the Substance Abuse and Mental Health Services Administration (SAMHSA). The HCS sought to test the effectiveness of an intervention for reducing opioid overdose deaths in 67 rural (n=29) and urban (n=38) communities heavily impacted by the opioid crisis across 4 states (KY, MA, NY, and OH) [8]. The HCS tested the Communities That HEAL (CTH) intervention, which deployed a community-engaged, data-driven process and tailored communication campaigns to assist communities in adopting and implementing evidence-based practices (EBPs) to reduce opioid fatalities [8]. Communities were randomized to either receive the CTH first (wave 1; n=34) or serve as the wait-list control group (wave 2; n=33) to receive the CTH after the HCS comparison period was completed [8]. Randomization only occurred at the community level in the main study, where communities were selected as part of the intervention group or wait-list control group. There was no randomization of the observational data reported in this paper. Complicated by the COVID-19 pandemic, an extreme rise of fentanyl in the illicit drug supply and the limited measurement period, the intervention did not significantly reduce the opioid overdose death rate in intervention communities [14]. However, an estimated total of 483 deaths were averted in the intervention arm (wave 1) compared to the control arm (wave 2).

The HCS communication campaigns aimed to support the adoption of EBPs (1) by improving awareness of and access to naloxone (an overdose reversal medication) and MOUD, (2) by driving demand for these EBPs, and (3) by reducing stigma, a key barrier to treatment initiation and recovery for individuals with substance use disorders [8]. A workgroup of communication experts from the research sites (Boston Medical Center, Columbia University, University of Kentucky, and The Ohio State University), the HCS Data Coordinating Center (Research Triangle Institute International), the National Institute on Drug Abuse, SAMHSA, and Oak Ridge Associated Universities (ORAU), an outside consultant on the study, conducted formative research and developed a standardized strategy (ie, topics, objectives, and priority audiences) and approach for the campaigns (ie, activities that shared messages with priority audiences). Priority audiences across all 4 sites included local community leaders and influencers, people at risk for an overdose or with OUD, and family and friends of those at risk. There were options at each site for crafting community-tailored messages (ie, including local photos), but all messages contained similar calls to action (eg, Carry naloxone. Save a life).

### Objectives

The development, dissemination, and monitoring of HCS campaigns created an opportunity for a more robust analysis of the performance of health communication campaigns, particularly those focused on OUD. Campaign development also involved in-depth formative research conducted by communications experts within multiple sectors, which is considered a best practice but is sometimes limited due to time, financial, and other resource constraints [15]. Thus, the current research effort adds to the existing literature on the effectiveness of health communication campaigns, expanding beyond smaller-scale OUD campaign message testing studies [11] and accounting for the exposure and engagement outcomes associated with the implementation of multigeographical site and multicommunication platform OUD campaigns [10]. Considering the above factors, we planned our assessment around 2 key campaign flight components: the exposure and engagement outcomes associated with specific tactics used in standard or template-based message dissemination (study 1) and the exposure and engagement outcomes associated with specific features of customizable, community-tailored campaign messages (study 2).

Considering this, the following research questions (RQs) were posed for the HCS tactics dataset (study 1):

- RQ1: How did click-through rate (CTR) differ based on the type of advertising method (social media and banner or display)?
- RQ2: How did CTR for social media advertisements differ across states (KY, MA, NY, and OH) and community types (urban and rural)?
- RQ3: How did CTR for banner or display advertisements differ across states (KY, MA, NY, and OH) and community types (urban and rural)?
- RQ4: How did cost-per-click (CPC) differ based on the type of advertising method (social media and banner or display)?
- RQ5: How did CPC for social media advertisements differ across states (KY, NY, and OH) and community types (urban and rural)?
- RQ6: How did CPC for banner or display advertisements differ across states (KY, NY, and OH) and community types (urban and rural)?

The following RQs were posed for the HCS community-tailored messages dataset (study 2):

- RQ1: How did CTR differ based on the type of advertising method (social media and banner or display)?
- RQ2: How did CTR for social media advertisements differ based on (1) approach (testimonial and information-based), (2) format (motion graphic or graphics interchange format [GIF], video, and static), (3) image (local and stock), (4) state (KY, MA, NY, and OH), and (5) community type (urban and rural)?
- RQ3: How did CTR for banner or display advertisements differ based on (1) approach (testimonial and information-based), (2) format (motion graphic or GIF, video, and static), (3) image (local and stock), (4) state (KY, MA, and OH), and (5) community type (urban and rural)?

## Methods

### Overview

The study start date was October 23, 2019, and the estimated study completion date was September 30, 2025.

The STROBE (Strengthening the Reporting of Observational Studies in Epidemiology) cross-sectional checklist was used in the writing of this manuscript [16]. This cross-sectional secondary analysis examines the relationships between different paid digital advertising strategies from a standard or template-based set of advertisements (study 1) and message content factors from a customizable, community-tailored set of advertisements (study 2) on engagement key performance indicators (KPIs) from communication campaigns developed for the HCS. KPIs are reported from each community and site as a whole, not as part of the original randomization of the communities into their respective intervention group or wait-list control group as part of the main study.

The outcomes presented here build upon a conceptual framework of KPIs that uses CTR and CPC as performance outcomes [1]. We compared 2 digital advertising methods—social media and banner or display—measuring cost and engagement KPIs. In study 1: HCS tactics, we examined the differences in CTR and CPC for each advertising method—social media or banner or display—across the states of KY, NY, and OH (Note: cost data from MA were not used because of inconsistencies, where multiple communities were grouped together for their advertising spend), and community type—urban and rural—separately. In Study 2: HCS community-tailored messages, no cost data were available, so only differences in CTR were examined within each advertising method (social media and banner or display). The dataset included detailed information about the characteristics of messages, so each analysis included one of the following independent variables: approach (testimonial and information-based), format (motion graphic or GIF, video, and static), image type (local and stock), state (KY, MA, OH, NY; Note: NY was excluded from banner or display analyses due to small sample size), and community type (urban and rural).

### Setting

The research site and ORAU staff collaborated closely with community coalitions and “champions” in each HCS community across KY, MA, NY, and OH to assess local needs and communication preferences, review local OUD data, and tailor campaign audience segments, materials, and dissemination strategies. Champions were coalition members who facilitated communication and activities between the coalitions, the HCS research teams, and partner organizations. HCS staff also provided technical assistance and training throughout the study to support coalitions in developing, implementing, and evaluating their campaigns. In total, the HCS designed, disseminated, and evaluated 264 local, community-tailored, and data-informed health communication messages about 5 different OUD topics from April 2020 to December 2023 (Table 1).

**Table 1.** Characteristics of HEALing^a^ Communities Study health communication campaign messages disseminated in wave 1 and wave 2 communities across KY, MA, NY, and OH.

Wave	Communities, n	Total campaigns, n	Topics	Objectives	Date range
1	33	165	Naloxone Stigma MOUD^b^ treatment Retention in MOUD treatment Community choice^c^	Increase demand for naloxone Increase access to and availability of naloxone Increase demand for, access to, and availability of MOUD Increase MOUD prescribing Improve acceptance and support for those in MOUD treatment Increase demand for, access to, and availability of MOUD Increase MOUD prescribing Increase retention in MOUD treatment for OUD^d^ Improve support for those in MOUD treatment	April 2020 to February 2022
2	33	99	Naloxone and fentanyl MOUD treatment and stigma Community choice^c^	Increase demand for naloxone Increase access to and availability of naloxone Increase knowledge about the prevalence and risk of fentanyl Increase demand for, access to, and availability of MOUD Increase MOUD prescribing Improve acceptance and support for those in MOUD treatment	November 2022 to December 2023

a HEAL: Helping to End Addiction Long-Term.

b MOUD: medication for opioid use disorder.

c “Community choice” indicates that communities had the option to repeat one of the prior campaigns based on their specific needs and preferences.

d OUD: opioid use disorder.

### Study 1: HCS Tactics Design

Coalitions used a study-provided template to identify paid and unpaid dissemination tactics they wished to implement for each HCS campaign. These plans included specifics about budget, types of advertising, and targeting parameters. Given the study campaigns aimed to drive traffic to community web pages containing OUD education and treatment information, all campaigns were optimized to generate “web page clicks” (engagement) over impressions (exposure). The study required all advertising to be, as much as possible, limited to the geographic boundaries or zip codes of each community. This requirement, and the relatively small budgets allocated for paid media, prevented communities from using “mass media” advertising, such as cable television. Research sites provided funding to communities for campaign dissemination. Most communities chose to purchase a mix of traditional (eg, print, local radio, local television, and billboards) and digital (eg, social media, digital display, and streaming television) advertising, although some communities did not purchase advertising for every campaign.

Most communities elected to target advertisements to similar audiences, including people with lived experience and community leaders. Communities used common digital media platforms and diverse audience targeting tactics to reach these audiences with advertisements; however, most communities elected to target campaign advertising using the demographics (eg, age and gender) associated with higher overdose deaths in each county and selected the media channels most used by those demographic groups. The second-most common targeting was behavioral, involving sharing advertisements with those who searched for or showed interest in drugs, alcohol, addiction, and treatment information.

To facilitate the purchasing and placement of paid advertisements, ORAU, on behalf of each research site, subcontracted with 4 media buyers, each with experience and expertise in 1 of the 4 state markets (KY, MA, NY, and OH). As part of their contracts, media buyers were required to provide communities and study staff with detailed paid advertising performance reports after each campaign, which formed the basis for the study data. The duration of communities’ paid advertising flights ranged from 5 to 133 days, with an average length of 59 days. The paid media costs per community per campaign ranged from US $75 to US $44,002, with an average cost of US $4630. Across all campaigns, the total impression volume was 85.9 million, and the total number of clicks to the HCS web pages was 369,679. Researchers could not estimate the number of specific audience members in each community (eg, number of people who use drugs in a specific community and number of community leaders).

### Study 2: HCS Community-Tailored Messages Design

HCS provided communities with a set of customizable social media and print materials (assets) and a website linking community members to additional information and local resources related to each campaign theme. The campaign assets were designed with “core” (unchangeable) messages and calls to action. Campaign assets could be locally tailored with options including imagery (local faces and places), content (medications, language, and personal testimonials or quotes), product (type of material, format, size, and design specifications), and destination (local provider, website, and phone number).

### Data Sources or Measurement

The method for collecting and analyzing paid media data across research sites involved a collaborative and iterative approach. Health communication experts from each site worked closely with media buyers to ensure strategic media placements and accurate data collection. Upon conclusion of the study, investigators sought to compare digital media performance across states and communities. To facilitate this process, a data manager developed a Microsoft Excel template that stored media buy method information (ie, display or banner and social media) and KPIs reported across all campaigns. KPIs included impressions, clicks, and media spend, from which CTR and CPC could be calculated (if not already provided in media buy reports). The template was refined through iterative feedback from staff at each site, ensuring that it was comprehensive and adaptable to the varied types of data collected across sites. Some KPIs, such as video completions, video completion rate, and reach, were not included in the final template, given that they were not consistently used or reported across states. This template, complemented by a codebook for data standardization, was shared across all sites. Study staff from each state entered media buy data from their community reports into this standard template, such that data could easily be combined and compared. The data manager reviewed data for errors, cleaned to address inconsistencies, and confirmed with site staff for accuracy.

### Variables

All KPIs used in these studies are reported based on the dates the campaigns were implemented in the HCS communities. As indicated earlier, the data were compiled, and the following variables were examined during the analysis.

In the HCS tactics dataset (study 1), CPC and CTR were used as dependent variables. CPC was calculated by dividing the total spend on the paid media tactic or platform within specific dates by the total number of clicks. CTR was calculated by dividing the total number of clicks by the total number of impressions. The independent variables were community type (urban and rural) and state (KY, NY, and OH; Note: MA was excluded from these analyses due to inconsistencies in their cost data, where multiple communities were grouped together for their advertising spend). Separate analyses were conducted for each advertising method (banner or display and social media). Banner or display advertising included cross-platform displays, device ID targeting, and native advertisements for seamless integration, along with search retargeting and in-app videos to engage users based on behavior and context. Strategies also leveraged standard display, preroll behavioral, and preroll contextual advertisements for targeted visibility. Social media advertising used Facebook for its broad reach and targeting capabilities, including boosted posts, and Instagram for visual engagement. Snapchat and TikTok targeted younger audiences with dynamic content, and YouTube provided a platform for video content.

In the HCS community-tailored messages dataset (study 2), CTR was the only dependent variable. No cost data were available, and as a result, CPC could not be calculated. Community type (urban and rural) and state (KY, MA, and OH; Note: NY was excluded from banner or display analyses due to small sample size) were used as independent variables. Additional message characteristics were also used as independent variables in this study. These characteristics included the approach for conveying the message (testimonial or information-based), the message format (motion graphic or GIF, video, or static image; Note: GIFs were excluded from social media analysis due to small sample size), and the image type (image of a local spokesperson or image of a stock person). Separate analyses were conducted for each advertising method (banner or display and social media).

### Statistical Methods

When analyzing the data, only cases with data for every variable of interest were included, resulting in different sample sizes for each analysis. Two datasets, HCS tactics (study 1) and HCS community-tailored messages (study 2), were analyzed. Data were not normalized by state or community for any of the analyses in either study. For Study 1, there were no missing data for any of the independent variables. When values for the dependent variables were unavailable, the corresponding media buy was removed from the analysis. Missing data for CTR represented 3.92% (18/459) of cases that met the inclusion criteria. Missing data for CPC represented 6.10% (28/459) of cases that met the inclusion criteria. For Study 2, there were no missing data on any of the independent variables. When values for the dependent variable were unavailable, the corresponding media buy was removed from the analysis. Missing data for CTR represented 0.99% (59/5913) of cases that met the inclusion criteria.

In the HCS tactics dataset (study 1), a Mann-Whitney *U* test was conducted to test differences in median percentage CTR (or median multiplied by 100) based on advertising method (RQ1). A Mann-Whitney *U* test was selected due to violations of normality, homogeneity, and linearity assumptions of parametric tests. Because differences were found, subsequent tests looked at each advertising method individually. Due to violations of normality and homogeneity assumptions in the data structure, Scheirer-Ray-Hare tests were conducted that looked at each advertising method individually (ie, one equation for social media [RQ2] and one for banner or display [RQ3]). Each test examined differences based on 2 independent variables, community type (rural vs urban) and state (KY, NY, OH). Dunn test with Bonferroni corrections was calculated to examine differences between pairs of states. The analytical process was duplicated with CPC as the dependent variable (RQ4, 5, and 6).

In the HCS community-tailored messages dataset (study 2), 3 statistical tests were conducted. CTR was the dependent variable for all tests. First, a Mann-Whitney *U* test was performed with the advertising method (social media vs banner or display) as the independent variable (RQ1). Because differences were found based on advertising method, 2 quantile regression equations were calculated that looked at each advertising method individually (ie, one equation for social media [RQ2] and one equation for banner or display [RQ3]). Quantile regression equations were used due to the nonnormal nature of the data. This test predicts the median percentage CTR (or median multiplied by 100), and when there were multiple levels of an independent variable, we used the level with the largest sample size as the reference group. In the social media regression equation (RQ2), the independent variables were state (KY, NY, OH, and MA), community type (rural vs urban), approach (testimonial or information-based), format (video or static image), and image type (image of a local spokesperson or image of a stock person). In the banner or display regression equation (RQ3), the independent variables were state (KY, OH, and MA), community type (rural vs urban), approach (testimonial or information-based), format (GIF, video, or static image), and image type (image of a local spokesperson or image of a stock person). No interaction terms were specified in either equation.

### Ethical Considerations

The HCS study protocol was approved by Advarra Inc (Pro00038088), a single institutional review board. The data analyzed here were observations of public behaviors and not primary analyses of the original study. Advarra Inc allowed for the secondary analyses conducted here to be conducted without additional consent. The data in this study were received from each state’s media buyer as aggregated, paid advertisement performance reports of KPIs not linked to any single individual. No individual participants were recruited, participated in, or compensated for this analysis, and as such, no identification of individual participants was possible (ie, data are deidentified).

## Results

### Study 1

#### HCS Tactics CTR

An overview of the state-level campaign tactics, audiences, and impressions can be seen in Table 2, and an overview of state-level campaign metric descriptives is reported in Table 3.

**Table 2.** Overview of state-level campaign impressions, target audiences, display or banner tactics, and social media tactics for the digital health communication campaigns of the HEALing^a^ Communities Study from April 2020 to December 2023.

State	Impressions, n	Audiences	Display or banner^b^	Social media
KY	31,122,896	Community leaders, people with lived experience^c^	Cross-platform display, device ID targeting, display, in-app video, native, preroll, search retargeting	Facebook, Instagram, Snapchat, YouTube
MA	18,555,039	Community leaders, people with lived experience^c^, health care providers	Cross-platform display, display, preroll	Facebook, Instagram
NY	27,535,463	Community leaders, people with lived experience^c^	Cross-platform display, preroll contextual	Facebook, Instagram, Snapchat, YouTube
OH	8,661,707	Community leaders, people with lived experience^c^, health care providers	Display, preroll behavioral	Facebook, Snapchat, TikTok, YouTube

a HEAL: Helping to End Addiction Long-Term.

b Tactics definitions: cross-platform display: advertisements displayed across multiple devices and platforms. Device ID targeting: advertisements targeted based on unique device identifiers. Display: banner or visual advertisements shown on websites or apps. In-app video: video advertisements embedded within apps. Native: advertisements that blend with the platform’s content and design. Preroll: video advertisements played before the main video content. Preroll behavioral: prevideo advertisements targeted based on user behavior patterns. Preroll contextual: prevideo advertisements tailored to the context of the content being viewed. Search retargeting: advertisements targeted to users based on their online search behavior.

c Including people who use drugs, people with opioid use disorder, and their loved ones.

**Table 3.** State-level campaign key performance indicators for the digital health communication campaigns of the HEALing^a^ Communities Study from April 2020 to December 2023.

State	Total impressions, n	Total clicks, n	Total cost (US $)	Click-through rate (%)	Cost-per-click (US $)
KY	31,122,896	90,843	223,330.30	0.291	2.46
MA	18,555,039	84,455	N/A^b^	0.455	N/A
NY	27,535,463	109,619	265,481.20	0.398	2.42
OH	8,661,707	84,762	92,598.90	0.978	1.09

a HEAL: Helping to End Addiction Long-Term.

b N/A: not applicable.

The Mann-Whitney *U* test resulted in a statistically significant difference in CTR based on the advertising method (*z*=9.27; *P*<.001; *r*=0.44, 95% CI 0.22%-0.44%; RQ1). Social media (mean 0.84%, SD 1.1%; median 0.54%, IQR 0.15%-1.03%) had a higher CTR when compared to banner or display (mean 0.21%, SD 0.27%; median 0.13%, IQR 0.08%-0.21%). Because the CTR was different for each advertising method, additional tests were conducted that examined each method separately based on state and community type. For social media (RQ2), the Scheirer-Ray-Hare test demonstrated that there was a statistically significant main effect for the state (*H*(2, 240)=27.16; *P*<.001). However, there was not a statistically significant main effect for community type (*H*(1, 240)=0.183; *P*=.67), and there was not a statistically significant interaction (*H*(2, 240)=0.163; *P*=.92). Dunn test with Bonferroni corrections found statistically significant pairwise differences between KY and OH (*z*=4.60; *P*<.001) and NY and OH (*z*=5.08; *P*<.001; Table 4). OH (mean 1.83%, SD 1.39%; median 1.35%) had a higher median CTR than both KY (mean 0.70%, SD 0.83%; median 0.52%) and NY (mean 0.67%, SD 0.95%; median 0.35%). For banner or display (RQ3), the Scheirer-Ray-Hare test demonstrated that there was not a statistically significant main effect for the state (*H*(2, 189)=5.78; *P*=.06) or community type (*H*(1, 189)=8.53; *P*=.003), nor was there a statistically significant interaction (*H*(2, 189)=4.16; *P*=.12). Dunn test with Bonferroni corrections found no statistically significant pairwise differences between states (Table 4).

**Table 4.** Click-through rate descriptives for social media and banner or display by state and community type for the digital health communication campaigns of the HEALing^a^ Communities Study from April 2020 to December 2023.

State and community	Social media			Banner or display		
	Advertisements, n	Mean (SD) (%)	Median (95% Mdn CI^b^) (%)	Advertisements, n	Mean (SD) (%)	Median (95% Mdn CI) (%)
KY						
Rural	40	0.70 (0.77)	0.52 (0.23-0.61)	66	0.28 (0.39)	0.14 (0.12-0.17)
Urban	48	0.70 (0.88)	0.53 (0.21-0.66)	71	0.17 (0.16)	0.12 (0.11-0.15)
Total	88	0.70 (0.83)	0.52 (0.28-0.62)	137	0.22 (0.30)	0.14 (0.12-0.15)
NY						
Rural	56	0.61 (0.53)	0.61 (0.24-0.69)	18	0.26 (0.24)	0.22 (0.17-0.27)
Urban	69	0.72 (1.18)	0.29 (0.23-0.50)	23	0.16 (0.14)	0.10 (0.08-0.13)
Total	125	0.67 (0.95)	0.35 (0.26-0.61)	41	0.20 (0.19)	0.15 (0.10-0.21)
OH						
Rural	19	2.08 (1.61)	2.00 (0.56-2.48)	11	0.13 (0.06)	0.11 (0.09-0.12)
Urban	14	1.49 (0.99)	1.30 (0.85-2.26)	6	0.07 (0.04)	0.05 (0.05-0.10)
Total	33	1.83 (1.39)	1.35 (0.75-2.45)	17	0.11 (0.06)	0.10 (0.05-0.12)
Total						
Rural	115	0.88 (1.02)	0.60 (0.46-0.65)	95	0.26 (0.34)	0.14 (0.13-0.17)
Urban	131	0.79 (1.08)	0.47 (0.29-0.63)	100	0.16 (0.15)	0.12 (0.10-0.14)
Total	246	0.84 (1.05)	0.54 (0.40-0.63)	195	0.21 (0.27)	0.13 (0.12-0.14)

a HEAL: Helping to End Addiction Long-Term.

b Mdn CI: median CI.

#### HCS Tactics CPC

The Mann-Whitney *U* test resulted in a statistically significant difference in CPC based on the advertising method (*z*=−7.47; *P*<.001; *r*=0.36; 95% CI US $2.47-$3.89; RQ4). Social media (mean US $5.59, SD US $8.95; median US $1.61, IQR US $0.98-$4.90) had a lower CPC when compared to banner or display (mean US $7.91, SD US $7.84%; median US $6.09, IQR US $3.79-$8.97). Because the CPC was different for each advertising method, additional tests were conducted that examined each method separately based on state and community type. For social media (RQ5), the Scheirer-Ray-Hare test demonstrated that there was a significant main effect for the state (*H*(2, 240)=12.05; *P*=.002), but no effect for community type (*H*(1, 240)=1.28; *P*=.26) nor an interaction (*H*(2, 240)=3.04; *P*=.22). Dunn test with Bonferroni corrections found significant pairwise differences between KY and OH (*z*=2.64; *P*=.03) and NY and OH (*z*=3.47; *P*=.002; Table 5). OH (mean US $2.50, SD US $5.68; median US $1.08) had a lower median CPC than both KY (mean US $4.03, SD US $5.19; median US $2.31) and NY (mean US $7.51, SD US $11.10; median US $2.04). For banner or display (RQ6), the Scheirer-Ray-Hare test demonstrated that there was a significant main effect for state (*H*(2, 189)=17.07; *P*<.001) and community type (*H*(1, 189)=6.99; *P*=.008), but not for the interaction (*H*(2, 189)=2.26; *P*=.32). Dunn test with Bonferroni corrections found significant pairwise differences between KY and OH (*z*=3.98; *P*<.001) and NY and OH (*z*=3.82; *P*<.001; Table 5). OH (mean US $15.50, SD US $12.50; median US $11.30) had a higher median CPC than both KY (mean US $7.51, SD US $7.46; median US $5.82) and NY (mean US $6.00, SD US $3.93; median US $5.48). Urban communities (mean US $8.83, SD US $8.26; median US $6.94) had a higher median CPC when compared to rural communities (mean US $6.94, SD US $7.29; median US $6.94). See Table 5 for a summary of CPC descriptives.

**Table 5.** Cost-per-click descriptives for social media and banner or display by state and community type for the digital health communication campaigns of the HEALing^a^ Communities Study from April 2020 to December 2023.

State and community	Social media			Banner or display		
	Advertisements, n	Mean (SD) (US $)	Median (95% Mdn CI^b^) (US $)	Advertisements, n	Mean (SD) (US $)	Median (95% Mdn CI) (US $)
KY						
Rural	40	4.24 (5.52)	2.56 (1.31‐3.89)	61	7.02 (8.18)	5.69 (4.02‐6.26)
Urban	48	3.86 (4.95)	2.31 (1.26‐3.13)	66	7.97 (6.77)	6.16 (5.14‐7.72)
Total	88	4.03 (5.19)	2.31 (1.35‐3.44)	127	7.51 (7.46)	5.82 (5.01‐6.56)
NY						
Rural	56	6.76 (10.80)	1.32 (1.14‐2.37)	18	4.33 (2.01)	4.19 (3.21‐5.17)
Urban	69	8.13 (11.30)	2.58 (1.74‐3.88)	23	7.31 (4.57)	7.49 (5.47‐8.91)
Total	125	7.51 (11.10)	2.04 (1.46‐2.77)	41	6.00 (3.93)	5.48 (4.04‐6.55)
OH						
Rural	19	2.93 (7.15)	1.15 (0.73‐1.16)	11	10.80 (6.01)	10.20 (5.31‐11.30)
Urban	14	1.90 (2.85)	0.82 (0.76‐1.58)	6	24.10 (17.10)	21.10 (9.85‐40.00)
Total	33	2.50 (5.68)	1.08 (0.81‐1.25)	17	15.50 (12.50)	11.30 (6.94‐13.90)
Total						
Rural	115	5.25 (8.80)	1.32 (1.20‐1.68)	90	6.94 (7.29)	5.52 (4.41‐6.23)
Urban	131	5.90 (9.10)	2.33 (1.46‐2.58)	95	8.83 (8.26)	6.94 (5.82‐7.88)
Total	246	5.59 (8.95)	1.61 (1.38‐2.33)	185	7.91 (7.84)	6.09 (5.48‐6.57)

a HEAL: Helping to End Addiction Long-Term.

b Mdn CI: median CI.

### Study 2

For the community-tailored messages dataset, the Mann-Whitney *U* test also resulted in a significant difference in CTR based on the advertising method (*z*=31.9; *P*<.001; *r*=0.42; 95% CI 0.18%-0.22%; RQ1). Social media (mean 0.84%, SD 1.40%; median 0.3%, IQR 0.12%-1.92%) had a higher CTR when compared to banner or display (mean 0.20%, SD 0.56%; median 0.08%, IQR 0.00%-0.19%). Because the CTR was different for each advertising method, additional tests were conducted that examined each method separately. The quantile regression equation that predicted CTR for social media advertisements (RQ2) found statistically significant predictors within each independent variable except for the approach (testimonial vs information-based). Urban communities, static format, and having a local spokesperson all produced a higher median CTR than their respective reference group (Tables6  7). Dunn test with Bonferroni corrections found statistically significant pairwise differences between all pairs of states: KY and MA (*z*=5.97; *P*<.001), KY and NY (*z*=3.90; *P*<.001), OH and KY (*z*=5.54; *P*<.001), OH and MA (*z*=7.85; *P*<.001), OH and NY (*z*=6.75; *P*<.001), and NY and MA (*z*=2.10; *P*=.04; Tables6  7).

**Table 6.** Click-through rate descriptives for social media by state, image type, format, community type, and approach for the digital health communication campaigns of the HEALing^a^ Communities Study from April 2020 to December 2023.

	Advertisements, n	Mean (SD) (%)	Median (95% Mdn CI^b^) (%)
State			
KY	2178	0.81 (1.26)	0.31 (0.27-0.34)
MA	140	0.42 (0.84)	0.12 (0.11-0.14)
NY	142	0.30 (0.23)	0.24 (0.21-0.29)
OH	137	2.31 (3.16)	1.26 (0.73-2.00)
Image			
Stock person	288	0.47 (0.91)	0.16 (0.12-0.24)
Local spokesperson	2309	0.88 (1.44)	0.31 (0.29-0.35)
Format			
Static	394	0.60 (1.02)	0.36 (0.24-0.45)
Video	2203	0.88 (1.45)	0.29 (0.27-0.32)
Community			
Urban	1468	0.94 (1.61)	0.33 (0.30-0.39)
Rural	1129	0.70 (1.06)	0.25 (0.22-0.30)
Approach			
Testimonial	2428	0.85 (1.43)	0.30 (0.27-0.33)
Information	169	0.60 (0.80)	0.37 (0.16-0.53)
Total	2597	0.84 (1.40)	0.30 (0.27-0.33)

a HEAL: Helping to End Addiction Long-Term.

b Mdn CI: median CI.

**Table 7.** Quantile regression coefficients for predicting click-through rate on social media using state, image type, format, community type, and approach for the digital health communication campaigns of the HEALing^a^ Communities Study from April 2020 to December 2023.

	Coefficient (SE) (%)	*t* test (*df*)	*P* value	95% CI (%)
Intercept	0.34 (0.03)	10.76 (8, 2589)	N/A^b^	0.24 to 0.40
State (reference=KY)				
MA	−0.24 (0.03)	−9.03 (3, 2589)	<.001	−0.31 to −0.16
NY	−0.06 (0.03)	−2.24 (3, 2589)	.02	−0.10 to −0.04
OH	1.00 (0.35)	2.89 (3, 2589)	.003	0.50 to 1.59
Image (reference=stock person)				
Local spokesperson	0.28 (0.10)	2.73 (1, 2589)	.01	0.21 to 0.43
Community (reference=urban)				
Rural	−0.08 (0.03)	−2.76 (1, 2589)	.01	−0.11 to −0.04
Format (reference=static)				
Video	−0.29 (0.10)	−2.88 (1, 2589)	.004	−0.39 to −0.25
Approach (reference=testimonial)				
Information	0.04 (0.04)	1.03 (1, 2589)	.31	−0.01 to 0.09

a HEAL: Helping to End Addiction Long-Term.

b N/A: not applicable.

The quantile regression equation predicting the median CTR for display or banner advertisements found (RQ3) statistically significant predictors for image (stock person vs local spokesperson) and format (static vs motion graphic or video). Static advertisements and advertisements with an image of a local spokesperson had a higher median CTR (Tables8  9). Dunn test with Bonferroni corrections did not find any statistically significant pairwise differences between any pairs of states, and the only statistically significant pairwise difference based on format was between static advertisements and video advertisements (*z*=2.38; *P*=.02).

**Table 8.** Click-through rate descriptives for display by state, image type, format, community type, and approach for the digital health communication campaigns of the HEALing^a^ Communities Study from April 2020 to December 2023.

	Advertisements, n	Mean (SD) (%)	Median (95% Mdn CI^b^) (%)
State			
KY	2558	0.21 (0.60)	0.08 (0.07-0.09)
MA	639	0.17 (0.35)	0.09 (0.08-0.09)
OH	119	0.26 (0.59)	0.11 (0.09-0.12)
Image			
Stock person	2230	0.19 (0.59)	0.07 (0.07-0.08)
Local spokesperson	1086	0.23 (0.48)	0.12 (0.10-0.13)
Format			
Static	1931	0.21 (0.63)	0.09 (0.08-0.09)
Motion graphic or GIF^c^	942	0.15 (0.18)	0.08 (0.06-0.11)
Video	443	0.29 (0.71)	0.06 (0.03-0.07)
Community			
Urban	1899	0.20 (0.50)	0.08 (0.08-0.09)
Rural	1417	0.21 (0.63)	0.08 (0.07-0.09)
Approach			
Testimonial	2771	0.21 (0.60)	0.08 (0.07-0.08)
Information	545	0.17 (0.25)	0.10 (0.09-0.11)
Total	3316	0.20 (0.56)	0.08 (0.08-0.09)

a HEAL: Helping to End Addiction Long-Term.

b Mdn CI: median CI.

c GIF: graphics interchange format.

**Table 9.** Quantile regression coefficients for predicting click-through rate on banner or display using state, image type, format, community type, and approach for the digital health communication campaigns of the HEALing^a^ Communities Study from April 2020 to December 2023.

	Coefficient (SE) (%)	*t* test (*df*)	*P* value	95% CI (%)
Intercept	0.08 (0.00)	21.66 (8, 3308)	<.001	0.07 to 0.08
State (reference=KY)				
MA	0.00 (0.01)	−0.18 (2, 3308)	.86	−0.01 to 0.02
OH	0.01 (0.02)	0.61 (2, 3308)	.54	0.02 to 0.06
Image (reference=stock person)				
Local spokesperson	0.09 (0.01)	11.27 (1, 3308)	<.001	0.07 to 0.11
Community (reference=urban)				
Rural	−0.01 (0.01)	−1.69 (1, 3308)	.09	−0.02 to 0.00
Format (reference=static)				
Motion graphic or GIF^b^	−0.04 (0.01)	−4.99 (2, 3308)	<.001	−0.05 to −0.02
Video	−0.12 (0.01)	−8.59 (2, 3308)	<.001	−0.14 to −0.09
Approach (reference=testimonial)				
Information	0.01 (0.01)	0.63 (1, 3308)	.53	−0.01 to 0.02

a HEAL: Helping to End Addiction Long-Term.

b GIF: graphics interchange format.

## Discussion

### Key Results

This paper assessed 2 key health communication campaign flight components: the exposure and engagement outcomes associated with specific tactics used in standard or template-based message dissemination (study 1) and the exposure and engagement outcomes associated with specific features of customizable, community-tailored campaign messages (study 2). In Study 1 (standard or template-based messages), social media had a higher CTR compared to banner or display (RQ1). Social media had a statistically significant main effect for state, but not for community type (RQ2), where OH had the highest CTR. There were no significant differences based on state or community type for display or banner (RQ3). Social media had a lower CPC compared to banner or display (RQ4). Social media also had a statistically significant main effect for state, but not community type, where OH had the lowest CPC (RQ5). Banner or display had a significant main effect for state and community type, where OH and urban communities had the highest CPC (RQ6). In Study 2 (community-tailored messages), social media also had higher CTR compared to banner or display (RQ1). For social media, advertisements in static format, advertisements featuring local spokespersons, advertisements in OH, and advertisements in urban communities had the highest CTR. There were no significant differences based on approach, that is, testimonial or information-based (RQ2). For display or banner, static advertisements and advertisements featuring local spokespersons had the highest CTR. Approach, state, and community type differences were not significant (RQ3).

### Interpretation

The results suggest that social media advertisements outperformed banner or display advertisements on CTR and CPC KPIs in the context of health communication campaigns intended to encourage information-seeking via click-throughs to website landing pages. However, these rates and costs varied as a function of the state where the campaign took place, the type of image used in the advertisement, the community type, and the format of the advertisement, suggesting that these contextual factors play a key role in the performance of digital campaigns.

Although means can be highly variable, median CPC was consistently lower for social media advertisements compared to banner or display advertisements; the extent varied by state, with OH having a much lower median CPC for social media compared to KY and NY. CTR differences by state were not simply attributable to the total dollar amount spent on media buys at each site but reflected differences in actual CTR. Social media CTR was similar for KY and NY but was higher in OH, which also had the best-performing CPC. There were no significant pairwise differences in the banner or display CTR between states, although the overall main effect for the state was significant and, descriptively, KY and NY CTR were similar while OH CTR was lower.

The differences in CTR and CPC performance by state provide a cautionary tale for the health communication practitioner. One state (OH) performed very well in response to social media advertisement placements but not in banner or display advertisements. Another state (NY) performed better with banner or display advertisements. The design and content of these banner or display advertisements were similar across sites. Considering the resources often invested in digital health communication campaigns, pilot testing across regions is a recommended best practice to direct planners toward the most effective advertising methods [17].

There are also some important caveats regarding the better overall CTR and CPC performance for social media advertisements compared to banner or display advertisements. It is possible that the placement of the social media advertisements in these campaigns was more appropriate than the placement of the banner or display advertisements or that the message content and strategy were better suited to social media advertising [18]. Furthermore, the data here demonstrate that CTR and CPC are key criterion variables when the desired outcome is to drive targeted audiences to act by visiting a website landing page. However, if the targeted outcome is different, such as increased attention to or enhanced recall of specific health information, banner or display advertising may prove to be more efficient and effective. Past research demonstrates that advertising message complexity is a key factor in attention and attitudinal responses, where fewer message features result in more recognition, comprehension, recall, and positive attitudes [19,20].

The results associated with CTR as a function of urban versus rural community types varied across advertising methods. In Study 1, CTR for banner or display advertisements was higher in rural communities than in urban communities. In Study 2, CTR for social media advertisements was higher in urban communities than in rural communities. In general, banner or display advertisements reach a broader audience across the internet, while social media advertisements are targeted to specific social media networks (eg, Facebook or Instagram). Social media use is generally higher in urban areas, as internet access is more reliable [21]. It is possible that banner or display advertisements are more appropriate for reaching rural individuals because of their more expansive reach. This is because banner or display advertisements appear on a wider range of websites (eg, news and shopping) and are not specifically related to one’s browsing activity. Alternatively, social media advertisements are only displayed within the specific platform that one actively uses.

There were 2 additional message factors that emerged as significant in study 2: the type of individual featured in the advertisement (stock photo person and local spokesperson) and the format of the advertisement (static, motion graphic or GIF, and video). Advertisements featuring local spokespeople and static advertising formats produced higher CTR than their respective reference groups. Here, using photographs of real people from the HCS communities initiated more engagement with campaign messages than stock photography. The stock images may have been perceived as less authentic or more unnatural by audiences than the photographs featuring real community members [22].

Kite et al [23] caution against creating media campaigns that use inappropriate or low-quality creative messaging; using photographs of real people may be one way to increase the quality and authenticity of messages. Regarding the performance of static formatted messaging in these studies, it seems counterintuitive that a less attention-getting format would be more successful than other types. However, when the intended outcome is to communicate clear and simple messaging, static may be a preferred format. Fan et al [24] also found that static and video messaging can elicit similar emotional responses. Static content is also more affordable to place than motion graphics or GIFs and video formats, which benefits those managing campaigns with budget constraints.

One final message factor was examined in this research effort: message approach (testimonial vs information-based). In general, narrative content garners more attention than purely informational content. In the present studies and counter to prior research findings [25], testimonial approaches were not more effective than information-based approaches [24]. However, considering that the target audience for these campaigns was communities highly affected by the opioid crisis, issue fatigue may have influenced engagement (or lack thereof). Issue fatigue occurs when a topic is consistently and regularly repeated and can result in issue avoidance and the dismissal of information [26]. Capturing and understanding community attitudes at the start of health campaigns via surveys appears to be a key component of formative research before launch. Furthermore, assessing community perceptions of campaign message repetition mid-flight could lend insight into issue fatigue. Both practices are recommended for future studies.

### Limitations

There are several limitations to report with this study. First, inconsistencies in the cost data from MA prevented us from including it in the analyses for study 1. Although we could account for cost variables, there were different campaign budgets and media buyers for each site, which increased variability in the data as a whole. Second, although each RQ presented here assessed different categorical types of advertisements, the overall number of RQs increases the possibility of type I error. Third, CTR and CPC are key criterion variables in evaluating campaign performance, but there are many others not assessed here (eg, time spent on page) that could also contribute to our understanding of campaign engagement. To this point, postclick behavioral outcomes (ie, landing and subsequent action on the HCS website) were not reported here because the landing website was maintained and reviewed by a third-party data coordinating center that was unable to verify the data. Algorithmic changes across Google and Meta regarding the placement of the advertisements were also unaccounted for. Finally, community coalitions and champions steered the types of paid advertising selected for each campaign. While media buyers and consultants provided guidance on effective strategies, communities varied widely in the types of advertising used and the specific audience segments and targeting parameters they chose to receive those advertisements. This high degree of variability did not allow for comparisons by audience or targeting parameters (eg, specific geographic locations and behavioral interests), which, along with the advertisements themselves, are known to influence campaign performance. Further, many communities experimented with more novel forms of digital media advertising (eg, streaming television advertising and mobile app advertising), but these efforts were not large enough to allow for meaningful analysis or comparison.

### Generalizability

We hope the data presented here provide some guidance for health communication scholars and practitioners in their implementation of digital media campaigns, especially those associated with OUD. This paper is one of the first to report on the performance of OUD campaigns using CTR and CPC measures, demonstrating the utility of their inclusion in future campaign evaluations. To date, many local, state, federal, and nonprofit agencies are implementing media campaigns with little guidance for best practices.

### Conclusions

In all, we can conclude that social media advertisements generally outperform banner or display advertisements in health communication campaigns, but only when considering key contextual factors, such as geographical location and type, imaging used, and format of the advertisement. Furthermore, using median CPC as a comparison metric instead of mean CPC is an effective metric for explaining campaign performance. Health communication practitioners should use pilot testing before launching digital campaigns to guide advertising methods. Desired campaign outcomes (eg, visits to a website landing page vs increased awareness and/or enhanced recall of information) should also be considered when implementing campaign strategies, tactics, and messages.

## Abbreviations

CPC: cost-per-click
CTH: Communities That HEAL
CTR: click-through rate
EBP: evidence-based practice
GIF: graphics interchange format
HCS: HEALing Communities Study
HEAL: Helping to End Addiction Long-Term
KPI: key performance indicator
MOUD: medication for opioid use disorder
NIH: National Institutes of Health
ORAU: Oak Ridge Associated Universities
OUD: opioid use disorder
RQ: research question
SAMHSA: Substance Abuse and Mental Health Services Administration
STROBE: Strengthening the Reporting of Observational Studies in Epidemiology
